# Supplementary material for: Assessment of healthcare waste management practices and associated factors in Addis Ababa City Administration Public Health Facilities
Source: PLoS One. 2022 Nov 4;17(11):e0277209. doi: 10.1371/journal.pone.0277209 (PMC9635710; doi:10.1371/journal.pone.0277209)
Supplement: Attachment — Submitted filename: Response to Reviewer.docx [file pone.0277209.s001.docx]

**Response to Academic editor and reviewer**

Dear Academic editors and reviewers thank you for your constructive comments.

**Comments to the author part**

The conclusion part of both the abstract and the revised manuscript is corrected according to the comments given by the reviewer.

**Review comments to the author**

**Reviewer #1 comment**

**Abstract**

1. In the abstract, most points in abstract result part are amended to the quantitative description.
2. The conclusion part is included to the abstract.

**Introduction**

The background in the revised manuscript is corrected. The abstracts indicated by the reviewer included to this part.

**Material methods**

In the revised manuscript the study period, inclusion and exclusion criteria and ethical consideration are included.

**Results section**

As the comment by the reviewer the sub-headings are corrected to the revised manuscript.

The healthcare waste management for manager as the study showed there were a total of 636 respondents among these 104 were different professional managers’ work in the health care facilities. Based on this information in the revised manuscript the new table (Table No 6) included indicating the number of professional managers worked in the health care facilities.

As reviewer comment the author changed most of the words “of the respondents” to the revised manuscript.

**Discussion**

In the revised manuscript the discussion part is amended and used the articles suggested by the reviewer and made all parts quantitative.

**Reviewer #2**

The author used WHO health care waste management assessment tool for this study. The study participants were health care waste Handlers who worked in the health centers and hospitals. The health care waste handlers were different professional and ancillary staffs. The managers were different health professional and ancillary staffs worked in the hospital and health centers.

The narrative of table 5 is amended based on the participants were responded. The language for the narrative also amended to the revised manuscript. The discussion part also amended to the revised manuscript.

To note that the author followed the manuscript writing according to submission guideline PLOS ONE.

It is my pleasure to see your response soon.

Tadesse, Menelik Legesse
