# Supplementary material for: Assessment of healthcare waste management practices and associated factors in Addis Ababa City Administration Public Health Facilities
Source: PLoS One. 2022 Nov 4;17(11):e0277209. doi: 10.1371/journal.pone.0277209 (PMC9635710; doi:10.1371/journal.pone.0277209)
Supplement: Attachment — Submitted filename: Comments for Manuscript Number PONE-D-21-40419.docx [file pone.0277209.s002.docx]

Comments for Manuscript Number:  PONE-D-21-40419

*Title:*  Assessment of healthcare waste management practices and associated factors in Addis Ababa City Administration Public Health Facilities.

*General remarks:*

Medical infectious waste management is not a new issue, even in Ethiopia. There have been several papers on HCW management in Ethiopia in the past 10 years. The difference of this paper is to reveal some specific data obtained from the study of 15 Public health centers and 3 hospitals in Addis Ababa city, Ethiopia.

The paper evaluated the rate of separation at source, on-site treatment of healthcare waste, and safety in healthcare management among healthcare centers and hospitals.

Besides, there are some comments as below:

- The manuscript needs to add more related papers that were previously studied in Ethiopia such as Yazie et al. BMC Res Notes (2019) 12:285 <https://doi.org/10.1186/s13104-019-4316-y>; Debere, M.K., Gelaye, K.A., Alamdo, A.G. et al. Assessment of the health care waste generation rates and its management system in hospitals of Addis Ababa, Ethiopia, 2011. BMC Public Health 13, 28 (2013). <https://doi.org/10.1186/1471-2458-13-28> or compared with similar studies but for private hospitals such as https://doi.org/10.1177%2F11786302211073383.
- The result discussion from Tables 1-4 in HCW management practice in page 14 is quite boring, repeated, and needs to be compared with the previous studies.
- The title HCW management in page 19 is repeated and needs to change to be appropriate with the content.
- The titles of Table 5, Table 7, and Table 12 should be more distinguished, at present, they are similar.
- The discussion from page 20 to page 34 needs to provide more comparison with similar studies in Ethiopia.
- Some Figures which have content similar to tables should be omitted (use either Table or Figure).

*Conclusion:* The paper had good information, however, it presents more like a report. The statistical analysis was presented too modest.

🡪 Accepted with major changes.
