# Supplementary material for: Assessment of healthcare waste management practices and associated factors in Addis Ababa City Administration Public Health Facilities
Source: PLoS One. 2022 Nov 4;17(11):e0277209. doi: 10.1371/journal.pone.0277209 (PMC9635710; doi:10.1371/journal.pone.0277209)
Supplement: Attachment — Submitted filename: Response to Reviewer.docx [file pone.0277209.s003.docx]

**Response to Academic editor and reviewer**

Dear Academic editors and reviewers thank you for your constructive comments.

**Review comments to the author**

**As per the Reviewer #1 comment**

-the author enriching the discussion part by 5 new journals recommended by the reviewer.

**As per the Reviewer #3 comment**

- the author amended the result discussion from table 1 to 4.

-the author changed the sub-title HCW management from page 19 to HCW management for managers

- the title of the table 7, 8, 9, 10, 11 and 13

- table 12 is merged with table 7

- the discussion part is amended by different 5 journals

To note that the author followed the manuscript writing according to submission guideline PLOS ONE.

It is my pleasure to see your response soon.

Tadesse, Menelik Legesse
